# Postpartum C-Reactive Protein: A limited value to detect infection or inflammation

**Published:** 2020-01-24

**Authors:** K Mertens *, J Muys *, Y Jacquemyn *

**Affiliations:** Department of Obstetrics, KLINA Hospital, Brasschaat, Belgium;; Department of Obstetrics and Gynaecology, Antwerp University UA ASTARC and Antwerp University Hospital UZA, Edegem, Belgium;; Department of Global Health, Antwerp University UA, Edegem, Belgium.

**Keywords:** CRP, C-reactive protein, endometritis, epidural anesthesia, infection, postpartum

## Abstract

**Background:**

During pregnancy the maternal immune system adjusts to preserve the foetoplacental unit. These adjustments lead to an increase in CRP, continuing into the postpartum. The objective of this study was to determineantepartal, peripartal and postpartal factors associated with an elevated CRP on the second postpartum day.

**Methods:**

A retrospective quantitative, monocentric file analysis in which antepartal, peripartal and postpartal factors were collected from a convenience sample was performed. On the second day postpartum CRP was taken according to local protocol. Uni- and multi-variate analysis was performed to determine factors that are related to postpartum level of CRP. The total sample size consisted of 1400 patients.

**Results:**

Multiple regression analysis indicated 11 factors related to increased CRP on the second day postpartum: gestational age (p=0.002), maternal blood leukocyte count on day 2 postpartum (p<0.001), artificial rupture of the membranes (p<0.001), fever during labor (p<0.001), indwelling urinary catheter (p=0.008), epidural anesthesia (p<0.001), fetal scalp electrode (p<0.001), primary planned caesarean (p=0.019), secondary caesarean h (p<0.001), formula feeding (p=0.030) and fever during postpartum (p=0.001).

**Conclusion:**

This research indicates that many antepartal, peripartal and postpartal factors are related to high postpartum CRP. CRP can not be used as a screening test test in the postpartum to discriminate between normal and pathologic inflammatory/infectious changes.

## Introduction

The acute phase protein, C-Reactive Protein (CRP), is a non-specific inflammatory marker related to both acute and chronic inflammation including infection, autoimmune reactions, malignancy, traumatic tissue damage and necrosis. Six to ten hours after the initial event, CRP rise becomes apparent in peripheral blood, often before clinical symptoms are present, resulting in a peak value at 48 hours and a rapid decline due to a brief half-life of 24 hours after resolution of the inflammatory process. CRP is in clinical practice often used as an infection or inflammation screening test. Serum concentrations of CRP in pregnancy are elevated above non pregnant values, the mechanism of this rise is unknown; the increase persists in the postpartum period (Skarżyńska et al., 2018).

Several factors have been linked to differences in postpartum CRP level including formula versus breastfeeding, vaginal versus caesarean delivery, gestational diabetes and hypertension. Relevant studies are mostly limited by small sample size and have shown conflicting results (De Meeus et al., 1998; Groer et al., 2005; Fetherston et al., 2006; Erkaya et al., 2014; Ozarda et al., 2014; Udenze et al., 2015). Therefore, the question remains which of these factors cause a significant increase in CRP and what role CRP plays as an inflammatory/infection parameter in the immediate postpartum to discriminate between physiologic changes and pathologic processes such as endomyometritis?

The goal of this study is to analize which antepartal, peripartal and postpartal factors are associated with an increase of CRP on the second day postpartum and which factors, together with CRP, predict infections in the postpartum.

## Materials and methods

A quantitative, monocentric cohort study was set up at the University Hospital of Antwerp UZA. The convenience sample of the study consisted of patients who delivered a live-born infant in the period between 1 September 2011 and 1 September 2015. On the second day after delivery (delivery = day 0) routinely blood was taken according to the existing local protocol, routine CRP testing was introduced at the end of the previous century without strong evidence. Serum level of CRP was determined by nefelometrics (Demension Vista 1500 System Siemens REF K7032).

Approval of the Antwerp University Hospital Ethics Committee was obtained under Belgian registration number (B30020162994) and it was approved to consult the Electronic Medical File (EMF) and the Electronical Nursing File (ENF) and use the medical data. Since it was a retrospective file analysis and there was no contact with the patients, an informed consent was not required. Anonymity was guaranteed by removing all identification from the medical data used in the database.

Inclusion criterium was delivery of a live fetus (no limitation on gestational age) in the hospital. Exclusion criteria were labor followed at home by a midwife, home birth followed by transport to the hospital, intrauterine fetal death, fever on admission (≥38.0°C) and suspicion or presence of chorioamnionitis on admission.

Collected data included maternal characteristics: maternal age, parity, gestational age, twin pregnancy, serological status for HIV, hepatitis B, hepatitis C, Body Mass Index (BMI), factors concerning labor: number of vaginal examinations, spontaneous or artifical rupture of membranes, hours between rupture of membranes and fetal expulsion, fever during labor (≥38.0°c sublingually measured), number of urinary catheterisations, presence of an indwelling urinary catheter, GBS (Group B streptococci) carriership, GBS antibiotic prophylaxis, type of antibiotics, number of administered doses of antibiotics, epidural analgesia, white blood cell count on the day of delivery, presence of an intravenous line, fetal scalp electrode, balloon induction of labor, vaginal administration of prostaglandin gel (Prepidil ® ), spontaneous versus induced labor, hypertensive problems (pre-existing hypertension, gestational hypertension, pre-eclampsia, eclampsia, HELLP syndrome (hemolysis, elevated liver tests and low platelets), diabetic problems (gestational diabetes, type 1 diabetes mellitus, type 2 diabetes mellitus), time between labor ward admission and delivery. Analysed intrapartum factors included: spontaneous versus assisted vaginal delivery, primary planned caesarean, secondary caesarean after labor, perineal status (intact, episiotomy, first, second, third, fourth degree perineal laceration), spontaneous placental expulsion versus manual removal of placenta; and finally the postpartum factors: breastfeeding or formula feeding, fever (>38.0°C sublingually measured), clinical diagnosis of endomyometritis (as per the interpretation of the clinician based on fever > 38°C, foul smelling discharge and uterine tenderness), wound infection (perineal or caesarean scar), phlebitis (based on clinical description of red swollen painfull venous region), cystitis (painfull frequent urination and culture proven > 105 colony forming units on a catheterized urine sample), postpartum administration of antibiotics, type of antibiotics and number of administered doses, leucocyte count determined on the second day of the postpartum and CRP determined on the second day of the postpartum. The correctness of the data collection was determined on the basis of double checking samples, three samples per 100 patients.

The collected data was analyzed by SPSS 24. Analysis was started with the drafting of frequency tables at which the continuous variables were expressed in mean ± SD (standard deviation) and discontinuous variables in valid percentage. Normal distribution of the sample was checked with Kolmogorov-Smirnov test. Spearman’s test was applied to study relations between ratio factors and CRP. For nominal factors first the difference in CRP level was calculated between groups using Kruskal-Wallis or Mann-Withney Test, significance was accepted at p<0.05. Then, pairwise comparison indicated the group in which the difference was situated in order to create dummies. Finally, univariate linear regression was used to study relations between nominal variables and CRP (significance accepted at p<0.05).

The factors related to a significant increase of CRP were included in multiple linear regression analysis with the aim to build an explanatory model for which factors explain a significant increase in CRP on the second day postpartum, significance accepted at p<0.05.

Prediction models were developed for different types of postpartum infections (endomyometritis, wound infection, cystitis) and phlebitis as a non infectious inflammatory process. The relation between nominal factors and different types of infections were calculated by Cramer’s V (p<0.05). The relationship between ratio factors and the different types of infections was calculated by Spearman’s test (p<0.05). Logistic regression was planned if data allowed this.

## Results

The total research population (N=1400) had an average age of 30.2 years (SD±5.0) and an average BMI of 24.07 (SD±4.66). Maternal characteristics are outlined in Table I. Kolmogorov-Smirnov testing indicated a non-parametric distribution of the population for maternal age, parity, gestational age, BMI and CRP level, an overview is presented in Table II. Median parity was 2, meaning that delivery of a second child was the most prevalent. Mean gestational age was 37.7 weeks (SD±3.4). Half of the population was admitted in spontaneous labor (n=729) with an average stay of nine hours (8.5h;SD±8.6) at the delivery ward. In 40 % membranes ruptured spontaneously with an average duration from rupture till childbirth of 4.9 hours (SD±13.3). Parturients had a median of 4 vaginal examinations, 40 % had at least one urinary catheterisation, indwelling catheters were exclusively placed in case of caesarean section (n=591, 42.2%). GBS was present in 15.3% (n=212), in 14.9% (n=206) carrierstatus was unknown. A large majority was breastfeeding (81.0%).

**Table I:** — Maternal Characteristics.

Maternal characteristics (N=1400)	Mean (±SD) - %
Age	30,20 (±5,00)
Parity	1,90 (±1,11)
Gestational age	37,76 (±3,39)
BMI	24,07 (±4,66)
Twin (n=62)	4,4%
Infection	
1. HIV (n=18)	1,3%
2. Hepatitis B (n=3)	0,2%
3. Hepatitis C (n=4)	0,3%
5. HIV + Hepatitis C (n=1)	0,1%
6. Others (n=8)	0,6%

Abbreviations: BMI: body mass index, HIV: human immune deficiency virus.

**Table II:** — Overview of antepartal, perpartal and postpartal factors in the population.

Antepartal factors (N=1400)	Mean (±SD) - %	Length of admission till childbirth	8,51 (±8,61)
Number of vaginal examinations	3,81 (±3,16)	Diabetes	
ROM		1. No diabetes (n=1322)	94,5%
1. Spontaneous (n=564)	40,9%	2. Gestational diabetes (n=55)	3,9%
2. Artificial (n=526)	38,1%	3. Diabetes type 1 (n=15)	1,1%
3. Artificial during caesarean (n=289)	21,0%	4. Diabetes type 2 (n=7)	0,5%
Interval between ROM and fetal expulsion (hours)	4,97 (±13,30)	Peripartal factors (N=1400)	Mean (±SD) - %
Fever (≥ 38,0°C) (n=39)	3,1%	Way of childbirth	
Number of urinary catheterisations	0,40 (±0,73)	1. Spontaneous delivery (n=809)	57,9%
Indwelling urinary catheter (n=448)	34,9%	2. Assisted vaginal delivery (n=148)	10,6%
GBS status		3. Planned caesarean (n=280)	20,0%
1. No carrier (n=968)	69,8%	4. Caesarean in labour (n=161)	11,5%
2. Carrier (n=212)	15,3%		
3. Unknown (n=206)	14,9%		
GBS prophylaxis (n=231)		Perineum	
when GBS positive or unknown	55,26%	1. Intact (n=692)	49,5%
Antibiotics (AB) labor/caesarean		2. First degree rupture (n=234)	16,7%
1. No antibiotics (n=792)	59,00%	3. Second degree rupture (n=212)	15,2%
2. Penicillin (n=222)	16,50%	4. Third degree rupture (n=10)	0,7%
3. Clindamycin (n=18)	1,30%	5. Fourth degree rupture (n=5)	0,4%
4. Cefuroxim (n=100)	7,40%	6. Episiotomy (n=238)	17,0%
5. Cefazolin (n=193)	14,40%	7. Episiotomy and rupture (n=7)	0,5%
6. Penicillin + cefuroxim (n=9)	0,70%	Postpartal factors (N=1400)	Mean (±SD) - %
7. Others (n=9)	0,70%	Feeding	
Number of doses of AB (n=1327)	0,56 (±0,90)	1. Breastfeeding (n=1129)	81,0%
Analgesia		2. Formula feeding (n=253)	18,2%
1. No analgesia (n=427)	30,7%	3. Breast- and formula feeding (n=11)	0,8%
2. Epidural analgesia (n=905)	65,1%	Fever (≥38,0°C) (n=19)	1,4%
3. General analgesia (n=48)	3,5%	Placental delivery	
4. Epidural + general (n=11)	0,8%	1. Spontaneous (n=939)	67,1%
WBC on day of delivery (.10^3/ml)	11,91 (±3,68)	2. Manual removal (n=461)	32,9%
Intravenous line (n=1130)	81,2%	Endomyometritis (n=6)	0,4%
Fetal scalp electrode (n=358)	25,6%	Wound infection (n=9)	0,6%
Situation on admission in the delivery ward		Phlebitis (n=1)	0,1%
1. Spontaneous labor (n=729)	52,2%	Cystitis (n=4)	0,3%
2. Induction with ballon and vaginal prostaglandin alone (n=214)	15,3%	Antibiotics postpartum (n=22)	1,6%
3. Induction with vaginal prostaglandin alone (n=78)	5,6%	Type antibiotics postpartum (n=1397)	
4. Previously hospitalized in MIC (n=232)	16,6%	1. amoxycilline-clavulanic acid (n=15)	1,1%
5. Planned caesarean (n=144)	10,3%	2. Penicillin (n=5)	0,4%
Blood pressure		3. Others (n=3)	0,2%
1. Normotensive (n=1280)	91,5%	WBC on day 2 after delivery (.10^3/ml)	11,38 (±3,10)
2. Essential hypertension (n=11)	0,8%	CRP day 2 postpartum (mg/L)	5,98 (±4,97)
3. Gestational hypertension (n=28)	2,0%		
4. Pre-eclampsia (n=54)	3,9%		
5. Eclampsia (n=2)	0,1%		
6. HELLP (n=24)	1,7%		

Abbreviations: ROM: rupture of membranes, WBC: white blood cell count, MIC: maternal intensive care, a dedicated ward for observation and treatment of problems during pregnancy e.g. preeclampsia, preterm labour. HELLP: hemolysis, elevated liver tests, low platelets, GBS: group B streptococci, AB: antibiotics.

During the study period 19 clinically diagnosed infections were registered: 6 endomyometritis, 9 wound infections, 4 cystitis). A single superficial phlebitis was noted.

**Figure 1:**
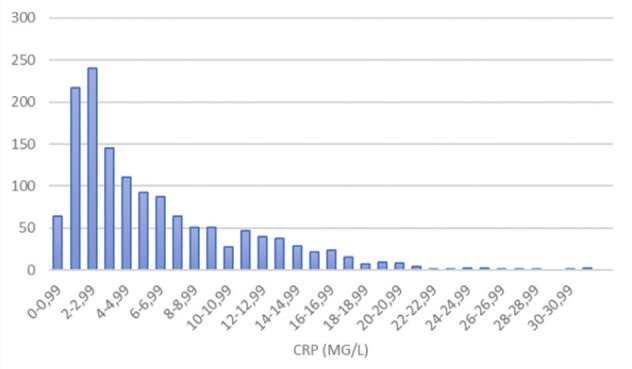
Distribution of CRP value in the research sample, vertical axis represents number of cases.

Postpartum CRP average level was 5.98 mg/L (SD±4.97); split up of levels according to clinical situation is presented in Table III. Figure 1 depicts the distribution of CRP values in the study population.

**Table III:** — Differences in CRP value within antepartal, perpartal and postpartal factors.

	mean CRP (mg/L) ±SD	p
ROM†		<0,001
1. Spontaneaous (n=564)	4,75 ±4,26	
2. Artificial (n=526)	5,13 ±4,47	
3. Artificial during cesarean (n=289)*	10,05 ±5,07	
Fever (≥ 38,0°C) (n=39)‡	9,69 ±5,39	<0,001
Indwelling catheter (n=448)‡	10,69 ±5,27	<0,001
GBS status†		<0,001
1. No carrier (n=968)	5,66 ±4,65	
2. Carrier (n=212)	5,23 ±4,91	
3. Unknown (n=206)*	8,06 ±5,77	
Analgesia†		<0,001
1. No analgesia (n=427)	2,87 ±1,90	
2. Epidural analgesia (n=905)*	7,28 ±5,27	
3. General analgesia (n=48)*	8,08 ±5,50	
4. Epidural and general (n=11)*	12,09 ±2,74	
Intravenous line (n=1130)‡	6,72 ±5,19	<0,001
Scalp electrode (n=358)‡	6,54 ±4,96	<0,001
Situation on admission in the delivery ward†		<0,001
1. Spontaneous labor (n=729)	4,58 ±3,96	
2. Induction with ballon and vaginal prostaglandin (n=214)	6,35 ±4,97	
3. Induction with vaginal prostaglandin alone (n=78)	5,36 ±4,59	
4. Previously hospitalized in MIC (n=232)	7,34 ±5,94	
5. Planned caesarean (n=144)*	10,57 ±4,59	
Blood pressure†		0,182
1. Normotensive (n=1280)	5,91 ±4,98	
2. Essential hypertension (n=11)	7,86 ±5,98	
3. Gestational hypertension (n=28)	5,91 ±4,92	
4. Pre-eclampsia (n=54)	6,92 ±4,68	
5. Eclampsia (n=2)	5,62 ±4,79	
6. HELLP (n=24)	6,91 ±4,17	
Diabetes†		0,017
1. No diabetes (n=1322)	5,88 ±4,91	
2. Gestational diabetes (n=55)*	7,94 ±5,95	
3. Diabetes type 1 (n=15)*	7,41 ±4,55	
4. Diabetes type 2 (n=7)*	6,96 ±4,43	
Delivery†		<0,001
1. Spontaneous vaginal (n=809)	3,45 ±2,55	
2. Assisted vaginal (n=148)	5,44 ±3,22	
3. Primary caesarean (n=280)*	9,92 ±5,09	
4. Secondary caesarean without labor (n=23)*	9,50 ±5,68	
5. Secondary caesarean with labor (n=138)*	12,87 ±4,99	
Perineum†		<0,001
1. Intact (n=692)*	8,11 ±5,80	
2. First degree laceration (n=234)	3,34 ±2,43	
3. Second degree laceration (n=212)	3,56 ±2,23	
4. Third degree laceration (n=10)	5,25 ±3,75	
5. Fourth degree laceration (n=5)	3,44 ±3,11	
6. Episiotomy (n=238)	4,61 ±2,96	
7. Episiotomy and laceration (n=7)	6,79 ±3,50	
Feeding†		0,030
1. Breastfeeding (n=1129)	5,79 ±4,84	
2. Formula feeding (n=253)*	6,75 ±5,36	
3. Breast and formula feeding (n=11)	6,18 ±5,10	
Fever postpartum (≥38,0°C) (n=19)‡	12,07 ±8,09	<0,001
Placenta‡		<0,001
1. Spontaneous expulsion (n=939)	3,83 ±3,03	
2. Manual removal (n=461)*	10,34 ±5,27	
Twin (n=62)‡	8,37 ±5,87	<0,001
Infection (n=34)‡	7,99 ±6,92	0,074
Endomyometritis (n=6)‡	11,10 ±7,32	0,024
Wound infection (n=9)‡	8,86 ±5,08	0,031
Phlebitis (n=1)‡	7,90	0,437
Cystitis (n=4)‡	11,80 ±5,19	0,024

Abbreviations: ROM: rupture of membranes, MIC: maternal intensive care, HELLP: hemolysis, elevated liver tests, low platelets.

* Group with significant elevation of CRP value; † Kruskal-Wallis; ‡ Mann-Whitney U.

Non-parametric test showed that any procedure resulted in a significant increase in CRP: patients not in labor who underwent primary caesarean (n=144) had a significant higher level of CRP (10.57 mg/L ± 4.59, p < 0.001) than patients who were admitted in spontaneous labor and delivered vaginally (4.58 mg/L ±3.96) and patients admitted for induction (balloon and prostaglandins: 6.35 mg/L±4.97;vaginal prostaglandins only: 5.36 mg/±4.59).

The use of analgesia (epidural analgesia and/ or general anesthesia) was related to significant difference in CRP levels (p<0.0001). Indwelling catheter (p<0.001), an intravenous line (p<0.001) or the use of a fetal scalp electrode (p<0.001) was also related to a significantly higher CRP. Vaginal birth (spontaneous or assisted) had a significantly lower CRP than caesarean section (p<0.001). Hypertensive disorders were not related to significant difference in CRP (p=0.182). CRP levels in patients with gestational diabetes (7.94 mg/L±5.95), diabetes type 1 (7.41 mg/L ± 4.55) and diabetes type 2 (6.96 mg/ L±4.43) were significantly higher than in non diabetic women (5.88 mg/L±4.91). Manual removal of the placenta (p<0.001), fever (p<0.001), endomyometritis (p= 0.024), wound infection (p=0.031) and cystitis (p=0.024) resulted in higher CRP level.

Univariate linear regression (Table IV) showed a significant relationship between an increased CRP and the following factors: fever during labor (p<0.001), indwelling catheter (p<0.001), GBS status unknown (p<0.001), anesthesia (p<0.001), intravenous line (p<0.001), scalp electrode (p=0.014), primary caesarean (p<0.001), diabetes (p<0.001), intact perineum (p<0.001), any caesarean (p<0.001), formula feeding (p=0.006), fever in the postpartum (p<0.001), manual removal (p<0.001), twin (p<0.001), endomyometritis (p=0.011) and cystitis (p=0.019). There was no significant relation between wound infection and CRP level (p=0.081).

**Table IV:** — Univariate and multivariate factors related to higher CRP on day 2 postpartum.

	Univariate			Multiple linear regression†		
Independent variables (N=1400)	rs		p	B	β	p
Age (n=1400)	0,012		0,651			
Parity (n=1393)	-0,178		<0,001	0,159	0,036	0,091
Gestational age (n=1389)	-0,119		<0,001	0,122	0,081	0,002
BMI (n=1208)°	0,163		<0,001			
Number of vaginal examinations (n=1246)°	-0,134		<0,001			
Interval between ROM and fetal expulsion (n=1256)	-0,028		0,329			
Number of bladder catheterisations (n=1251)	0,024		0,395			
WBC on day of delivery (n=1400)	0,008		0,774			
Duration admission till birth (n=1367)	-0,039		0,147			
WBC 2 postpartum (n=1400)	0,252		<0,001	0,323	0,200	<0,001
	R	R^2^				
Amnioyomy during caesarean (n=289)	0,418	0,175	<0,001	1,770	0,146	<0,001
Fever during labor (n=39)	0,127	0,016	<0,001	2,999	0,103	<0,001
Indwelling catheter (n=448)	0,652	0,425	<0,001	2,396	0,226	0,008
GBS unknown (n=206)	0,178	0,032	<0,001	0,233	0,017	0,435
Any analgesia (n=964)	0,418	0,174	<0,001	1,387	0,126	<0,001
Intravenous line (n=1130)	0,301	0,090	<0,001	0,235	0,018	0,524
Scalp electrode (n=358)	0,066	0,004	0,014	0,933	0,082	<0,001
Planned caesarean (n=144)	0,314	0,099	<0,001	1,084	0,068	0,019
Diabetes (n=77)	0,086	0,007	0,001	0,441	0,020	0,321
Intact perineum (n=692)	0,424	0,180	<0,001	0,167	0,017	0,556
Any caesarean (n=441)	0,658	0,434	<0,001	5,216	0,489	<0,001
Formula feeding (n=253)	0,074	0,006	0,006	0,551	0,043	0,030
Fever during postpartum (n=19)	0,144	0,021	<0,001	3,458	0,074	0,001
Manual removal of placenta (n=461)	0,616	0,380	<0,001	0,245	0,023	0,665
Twin (n=62)	0,104	0,011	<0,001	0,150	0,006	0,762
Endomyometritis (n=6)	0,068	0,005	0,011	1,145	0,013	0,524
Wound infection (n=9)	0,047	0,002	0,081	1,745	0,030	0,142
Cystitis (n=4)	0,063	0,004	0,019	4,583	0,026	0,197
				R^2^ = 0,551	Adjusted R^2^ = 0,543	

Abbreviations: R: correlation coefficient, R2 coefficient of detremination, RS: Spearman’s rank, B, ROM: rupture of membranes, BMI: body mass index, WBC: white blood cell count; GBS: group B streptococci.

°BMI and number of vaginal examinations were not further analyzed because of > 10% missing data.

Multiple linear regression demonstrated 11 factors related to rised CRP levels (Table IV). These factors were: gestational age (p=0.002), blood leucocyte count on day 2 (p<0.001), artificial rupture of the membranes during caesarean (p<0.001), fever during labor (p<0.001), indwelling catheter (p=0.008), anesthesia (p<0.001), scalp electrode (p<0.001), primary caesarean (p=0.019), any caesarean section as birth mode (p<0.001), formula feeding (p=0.030) and fever during postpartum (p=0.001).

No prediction model was built because of the low prevalence of endomyometritis (n=6), wound infection (n=9), phlebitis (n=1) and cystitis (n=4) on a sample size of 1400.

Univariate regression to find relations between endomyometritis, wound infection, phlebitis, cystitis and antepartal, perpartal and postpartal factors failed to find any significantly relation. As a result, no prediction model could be built.

Routine CRP testing after delivery was stopped in our hospital after this study.

## Discussion

This study shows that several antepartal, peripartal and postpartal factors are related with increased CRP level on the second day postpartum, including routinely and frequently performed procedures like amniotomy and epidural anesthesia. All factors are closely related to each other (e.g. urinary catheter and caesarean section and epidural analgesia).

Most of these relations do not come as a surprise, such as urinary catherisation and cystitis or an increasd inflammatory response after repeated vaginal examinations. Some are less evident, such as a lower CRP in case of breast feeding, a finding from our study that confirms previous research (Groer et al., 2005; 2013). There was no case of mastitis in this series, which is not surprising as mastitis is very rare in the first days after birth.

Contradictory results have been published concerning CRP and hypertensive disorders of pregnancy, we were not able to find a difference between normotensive and hypertensive women, as were Groer et al. (2013), in contrast Udenze et al. (2015) demonstrated a significantly higher CRP in pre-eclamptic women. Part of these contradictory results can be due to the fact that Udenze et al. (2015) only included severe pre-eclamptic patients (systolic pressure ≥160 mmHg and/or diastolic pressure ≥110 mmHg and 2+ proteinuria), whereas we included also mild and moderate cases. Different types of diabetes (gestational diabetes, diabetes type 1 and 2) were not related to higher CRP in our data, others did find such a correlation (Vrachnis et al., 2012a; 2012b), differences can be due to the obtained level of glycemic control or the relation with obesity: obesity being a well known associated factor for chronic inflammation. BMI and the number of vaginal examinations was not included in multiple regression analysis although univariate analysis showed a significant relation between these factors and the increase of CRP. This was done because of more than 10% loss of data. Christian and Porter (2014) showed that BMI and CRP level are linked which is confirmed in our research (r = 0.163; p < 0.001).

The major strength of our study is the large amount of included and non selected women, but several weaknesses should be considered including the retrospective nature of the study. Patients with preterm labor were included, when clinical chorioamnionitis (fever, maternal or fetal tachycardia, uterine tenderness) was not diagnosed by the gynaecologist in charge, although we realize that preterm labor is often related to subclinical chorioamniotic infection (Erdemir et al., 2013; Howman et al., 2012). Therefore, it is possible that patients with a subclinical chorio-amnionitis were included. In addition, stress and autoimmune diseases were not taken into account although they have an influence on CRP. At the same time analgesia with non steroidal antiinflammatory drugs can supress CRP levels, and ibuprofen is routinely administered according to hospital protocol after delivery.

The low percentage of the endomyometritis (0.4%), cystitis (0.3%), wound infection (0.6%) and phlebitis (0.1%) made it impossible to build a prediction model. The low percentage of postpartum infections is remarkable in comparison to numbers in literature (prevalence of endomyometritis is estimated 1.7% after planned caesarean, 3% after vaginal delivery and 11% in case of secondary caesarean). As this is a retrospective data collection some cases might be missed, on the other hand wound infection after caesarean is part of a systematic quality control system in our hospital and has been below 1% for several years before this study.

## Conclusions

High CRP in the postpartum is related to almost any procedure during labor, including artifical rupture of the membranes, more frequent vaginal examinations, epidural analgesia, fetal scalp electrode and also to postpartum factors such as bottle feeding. In a population with very low infectious postpartum complications we were not able to use CRP to predict endomyometritis, cystitis or wound infection. CRP can not be used as a screening test in the postpartum to discriminate between normal and pathologic inflammatory/infectious changes.
